# Intra-articular Steroid alone vs Hydrodilatation with intra-articular Steroid in Frozen Shoulder - A Randomised Control Trial

**DOI:** 10.5704/MOJ.2303.005

**Published:** 2023-03

**Authors:** S Swaroop, P Gupta, S Patnaik, SS Reddy

**Affiliations:** 1 Department of Orthopaedics, Siksha 'O' Anusandhan Institute of Medical Sciences and SUM Hospital, Bhubaneswar, India; 2 Department of Orthopaedics, Sandwell and West Birmingham Hospitals NHS Trust, West Bromwich, United Kingdom

**Keywords:** frozen shoulder, hydrodilatation shoulder, intra-articular steroids, adhesive capsulitis

## Abstract

**Introduction:**

Various non-operative treatment modalities have been advocated for a frozen shoulder. In the present study we compared the efficacy of single intra-articular steroid injection vs hydrodilatation with intra-articular steroids for frozen shoulder (FS) in the frozen phase.

**Materials and methods:**

This was a prospective, randomised control trial (RCT) done at a tertiary care centre. A total of 108 participants were randomised into two groups-one group received intra-articular steroid with hydrodilatation (HDS) and other group received intra-articular steroid injection only (S). Shoulder Pain and Disability Index (SPADI) scores were taken, and statistical analysis was done to measure the outcome at two weeks, six weeks and three-month intervals after the injection.

**Result:**

There was significant improvement in symptoms at each interval for both the groups (p=0.0). There was no statistically significant difference in the SPADI score between the two groups at two weeks post injection, however at six weeks (p=0.04) and 3 months (p=0.001) significant difference in the SPADI score was demonstrated with better scores in group S. The mean duration of analgesia required in group HDS was 5.17 days (S.D.=1.73) and for group S was 4.28 days (S.D.=1.01), with a statistical significance (p=0.002).

**Conclusion:**

Better clinical results were obtained at six weeks and three months with the group receiving corticosteroid only and also had a lesser requirement of analgesia post-intervention. Thus, intra-articular steroid injection only seems to be a more desirable method of management during the frozen phase of FS than that of hydrodilatation with intra-articular steroid injection.

## Introduction

Frozen shoulder (FS) is a self-limiting pathology of the shoulder joint causing restriction in both the active and passive range of movements associated with pain^1^. The condition results from progressive fibrosis and eventual contracture of the capsule of the glenohumeral joint, which causes pain and stiffness^2,3^.

The occurrence of this FS is 2-5% in the general population^4^. FS may persist for more than three years or may never resolve^5^. Conservative management includes physiotherapy, NSAIDS, Hydrodilatation (HD), intra-articular steroids, manipulation under anaesthesia, arthroscopic, or open capsular release^6^. Distension of the shoulder capsule with normal saline and steroid infusion has been suggested to give benefit by changing the biochemical properties of the capsule in FS^7^. A recent study and metanalysis have suggested that there are no added advantages of adding steroids to HD^8,9,10^. On the contrary, Ladermann *et al* and Catepano *et al* inferred from their study that the best conservative treatment for frozen shoulder is HD with steroids^11,12^.

Such conflicting conclusions by various authors have not led to any consensus. Thus, we conducted a study to find any difference in outcomes with the two different treatment modalities for FS. We hypothesised that there will be no difference in outcome when comparing the treatment of FS in patients receiving only intra-articular steroids (S) and another group receiving HD with steroids (HDS).

## Materials and Methods

This study aims to compare the outcome of treating FS with two different treatment modalities, (1) Hydrodilatation, (2) Intra-articular steroids. The primary outcome of the trial is comparing the functional outcome of the two modalities by SPADI score and the secondary outcome would be to look for any complications of the two treatment modalities. The diagnosis of FS was based on examination findings of restricted range of movement (ROM) on passive and active movements of the shoulder in at least two planes. Patients were examined by a senior orthopaedic surgeon who looked for restricted active and passive ROM < in at least 2 directions (abduction and forward flexion <100°, external rotation <20°, or internal rotation <L3)^13^. Radiological examination with a radiograph, AP, axillary views, and ultrasonography were done to rule out any alternative cause. This was a randomised prospective trial done at a tertiary care centre, Bhubaneswar from March 2021 to October 2021.

Patients with unilateral frozen shoulder of ages above 18 and below 70 years with symptoms more than three months but less than one year which has not improved with medications and physiotherapy were included in the study. Patients with diabetes, history of trauma to the shoulder, history of fracture or dislocation around the shoulder, rotator cuff pathology, inflammatory arthritis, thyroid disease, history of previous injections within three months, and cervical radiculopathy were excluded from the study. The study complied with the Helsinki Declaration as revised in 2013. Institutional board ethical clearance (IEC/IMS.SH/SOA/2021) was obtained, and all patients were included after taking informed written consent.

**Fig. 1:**
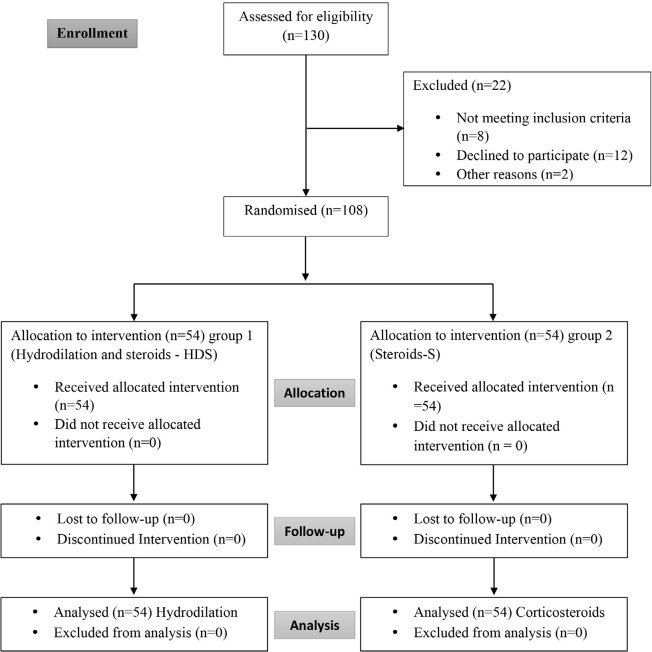
CONSORT flowchart diagram indicting patient flow.

A total of 108 participants who satisfied the inclusion criteria were enrolled by SS after consent for participation in the trial. Participants were randomised into two groups by the method of variable block randomisation using R software. CONSORT Flowchart (Fig. 1). The allocation ratio was 1:1, where the treatment modality was allocated by a third person who was not involved in the study by selecting sealed opaque randomly numbered envelopes. The investigator and participant was blinded to the intervention.

**Fig. 2:**
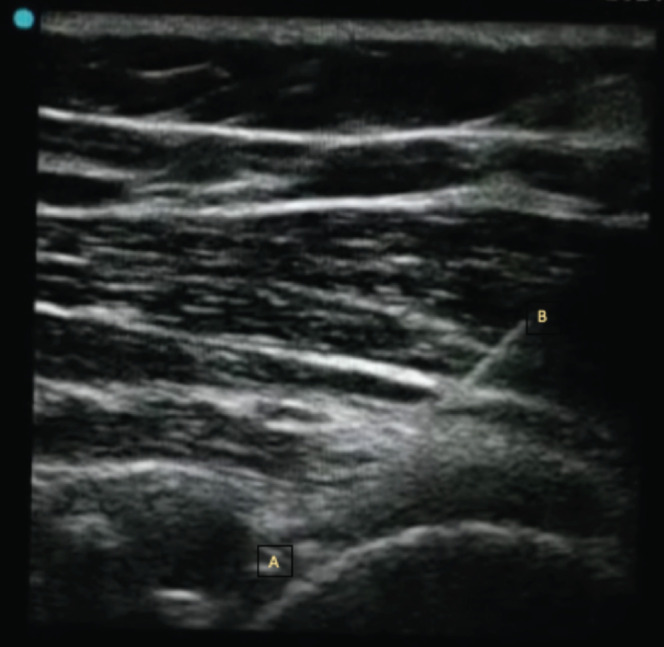
Hydrodilatation with guidance of ultrasound in the glenohumeral capsule by the posterior approach (transverse view), A: Glenohumeral joint, B: Injecting Needle.

Hydro dilatation was done under all aseptic measures, the interventional radiologist under ultrasound guidance and using the posterior approach injected into the joint capsule (Fig. 2), 4ml of 2% lignocaine, 4ml of 0.2% ropivacaine, 2ml of triamcinolone (40mg/ml) and filled to full with normal saline in a 50-cc syringe in HDS group. The fluid was injected under ultrasound guidance in the HDS group until the capsule was distended (until the axillary pouch appeared full) and resistance was felt taking care not to rupture the capsule. The amount of fluid required to dilate the capsule was recorded. Similarly, participants receiving only intra-articular steroids were given the injection under aseptic precautions with the guidance of ultrasound with 4ml of 2% lignocaine, 4ml of 0.2% ropivacaine, and 2ml (40mg/ml) of triamcinolone with a 22-gauge needle to the patients in S group.

All participants were assigned to an identical physiotherapy protocol: capsular stretching (day 1), modified sleeper’s stretch, and cross-body stretch with breathing, range of motion (ROM) exercise (day 1), pendulum exercise, and active assisted exercise (wand exercise). Isometric strengthening exercises were started on day 1, whereas isotonic concentric exercises were started on day 14. A moist heat pack was applied before starting exercise. The patients were given local ice packs along with NSAIDS (aceclofenac100mg and paracetamol 325mg twice daily) for 3 days and as and when required with subsequent days. They were followed-up subsequently with identical physiotherapy protocols and outcome measures were evaluated by SPADI^14^ score at two weeks, six weeks, and three-month intervals. Secondary outcome was the duration of analgesia (days) required by the participants post injection in both groups. Any adverse events of injection were also noted.

Sample size calculation in a previous study^15^, a difference in SPADI score of 10 points or more was chosen as a minimal clinically significant difference in shoulder pain and function. Published data on this point is rather limited, but for practical reasons, we adopted the same figure in our sample size calculations. With an alpha (p-value) of 5% and a power of 80%, this would result in a necessary sample size of 44 patients per group for a two-sided t-test. With a dropout rate of 20%, the sample was calculated to be 54 in each group.

We aimed at finding any statistically significant difference in the outcome of the two groups at two weeks, six weeks, and three months and the statistical difference between the days of analgesia required between the two groups. We applied the independent "t" test to analyse the difference in mean SPADI scores at the pre-intervention and post-intervention intervals and days of analgesia requirement. For statistical analysis, SPSS 22.0 software [IBM, Armonk, NY, USA] was used.

## Results

One hundred and eight patients were included in the study after considering the inclusion and exclusion criteria divided equally into both groups. The mean age in group HDS was 57.57 years (S.D.=8.53) and the mean age in group S was 58.13 years (S.D.=6.39), there was no statistically significant difference between the two groups in the mean age (p=0.3).

Moreover, the mean duration of symptoms was 3.56 months (S.D.=1.20) in group HDS and 3.50 months (S.D.=1.14) in group S. No significant difference was found between the mean duration of symptoms between the two groups (p=0.86). Following the injection, both groups received the same analgesia protocol, however, the mean duration of analgesia required in group HDS was 5.17 days (S.D.=1.73) and for group S was 4.28 days (S.D.=1.01), which is statistically significant (p=0.002) (Table I).

**Table I:** Age, duration of symptoms and use of analgesia in days compared between both the groups

	Group	N	Mean SPADI score (S.D.)	p-value
Age	HDS	54	57.57 (8.53)	p=0.3
	S	54	58.13 (6.39)	
Duration (Symptoms)	HDS	54	3.56 (1.20)	p=0.86
	S	54	3.50 (1.14)	
Days of analgesia (post intervention)	HDS	54	5.17 (1.73)	p=0.002
	S	54	4.28 (1.01)	

Abbreviations – SPADI: Shoulder Pain and Disability Index, S: Steroid only group, HDS: Hydrodilatation and steroids

After the intervention, both groups received the same analgesics and physiotherapy protocol and the SPADI score was analysed before the intervention, after two weeks, six weeks, and at three months' time after the intervention. On statistical analysis, there was a significant improvement in symptoms at each interval (p=0.0). No significant difference was found between the mean SPADI scores of the two groups before the intervention, indicating that we had similar scores in both groups before the intervention. When each group was compared at each interval after the intervention, it was found that there was no statistically significant difference in SPADI score between the two groups at two weeks after the injection, however, at 6 weeks (p=0.04) and 3 months (p=0.001) a significant difference in SPADI score was demonstrated with better scores in group S. Mean amount of fluid injected in HDS group was 18.4ml (S.D.=3.0). In the S group, the amount injected was 10ml for all participants. Two participants had an episode of flushing and one participant had severe pain for which the procedure was interrupted and competed after a few moments. No participant had any adverse reaction in the S group (Table II).

**Table II:** SPADI score at each time interval of follow-up in both the groups

Time interval	Group	N	Mean SPADI score	p-value
Pre-injection	HDS	54	71.56	p=0.589
	S	54	70.30	
2 weeks	HDS	54	44.56	p=0.431
	S	54	43.20	
6 weeks	HDS	54	31.02	p=0.04
	S	54	28.98	
3 months	HDS	54	23.11	p=0.001
	S	54	21.18	

Abbreviations – SPADI: Shoulder Pain and Disability Index, S: Steroid only group, HDS: Hydrodilatation and steroids

## Discussion

On comparing the results of both groups at three months, we found that any form of intra-articular steroid injection (with or without HD) resulted in significant improvement in pain and disability in patients suffering from FS. When we evaluated the SPADI score at two weeks, there was a significant improvement in both groups but there was no statistical difference between them. This may indicate that HD may not have any immediate additional advantage over intra-articular steroids, however, there was a significant improvement in the SPADI score in both groups suggesting that corticosteroids play a major role in reducing the symptoms of a patient with unresolved FS.

There have been numerous studies in the past which have supported HD along with corticosteroids, but the direct effect of hydro-dilation has been difficult to analyse^9,11,15-19^. In our study, we have tried to compare two groups, one with HD and steroids and the other with only steroids, to determine the advantage of adding HD to intra-articular steroid injection. Few comparative studies have found no benefit of HD combined with corticosteroids over corticosteroids alone^9,18^. A recent systematic review and meta-analysis by Saltychev *et al* also concluded that hydro-dilatation has a clinically insignificant effect in the treatment of FS^19^.

Our study has a larger sample size and we have included patients after three months of unresolved symptoms who were in their frozen stage. In our study, we found better functional outcomes at six weeks and three months in the group that received steroids only, suggesting no additional benefit of HD similar to the study by Paruthikunnan *et al*^8^. The reason HDS group showed inferior results may be due to the dilution of steroids and the capsular stretch causing inflammation^15^. Moreover, HD with steroid method may slow down the improvement by causing distension injury to the capsule and capsular stretch pain^9^. Thus, leading to statistically better results in the group of patients without HD at six weeks and three months of follow-up.

The mechanism of action of HD is supposed to either by stretching or rupturing the capsule, thus improving the glenohumeral mobility^15^. Few studies have used a large volume of injection leading to rupture of the capsule^15,17^. Capsule rupture leads to the expulsion of solution (including steroids) out of the joint leading to decreased efficacy of corticosteroids^18^. It was noteworthy in our study that after the intervention both groups received the same protocol of analgesics and physiotherapy, but the steroid group required analgesics for a lesser duration and the difference was statistically significant (p=0.002). The HDS group requiring more analgesics may be due to the distention effect of the capsule as we have avoided rupturing to achieve maximum distension of the capsule in our study.

In a study by Yoon *et al*^17^, the group with HD and corticosteroids had better outcome compared to the corticosteroid alone group at 1st month, 3rd month follow-up based on the visual analogy scale (VAS) score for pain and range of motion (ROM). This may be attributed to the fact that they received 3 injections one after the other and it would be clinically more relevant to differentiate between the two methods based on improvement after one injection. However, there was a clinically similar improvement when both groups were followed-up until six months, which proves corticosteroids may be the main benefactor in the overall treatment process and the initial HD effect is only short-lived. However, the process of HD by Yoon *et al* was until the rupture of the capsule and may be the reason for the results varying in our study.

The limitations of our study are that we have used only the SPADI score to evaluate the outcome. ROM and VAS score was not assessed as they may be very subjective measures. However, we used SPADI to measure day-to-day morbidity caused by FS. A three-month follow-up may warrant further evaluation, but FS being a self-limiting disease, a longer follow-up may not indicate the outcome of the intervention. We did not take into account participant comorbidities.

## Conclusion

Better clinical results were obtained at six weeks and three months with the group receiving corticosteroids only and had a lesser requirement of analgesia post-intervention. Thus, intra-articular steroid injection only seems to be a more desirable method of management during the frozen phase of FS than that of hydrodilatation with intra-articular steroid injection.
